# Egg incubation temperature differently affects female and male hatching dynamics and larval fitness in a leafhopper

**DOI:** 10.1002/ece3.89

**Published:** 2012-04

**Authors:** Julien Chuche, Denis Thiéry

**Affiliations:** INRA-ENITAB UMR 1065 Save Santé et Agroécologie du vignoble, INRA, BP 81, Institut des Sciences de la Vigne et du Vin33883 Villenave d’Ornon Cedex, France

**Keywords:** Grape, insect vector, operational sex ratio, protandry, *Scaphoideus titanus*, sex ratio

## Abstract

Temperature effects on ectotherms are widely studied particularly in insects. However, the life-history effects of temperature experienced during a window of embryonic development, that is egg stage, have rarely been considered. We simulated fluctuating temperatures and examined how this affects the operational sex ratio (OSR) of hatching as well as nymph and adult fitness in a leafhopper, *Scaphoideus titanus*. Specifically, after a warm or cold incubation we compared males and females hatching dynamics with their consequences on the sex ratio in the course of time, body size, weight, and developmental rate of the two populations, all reared on the same posthatching temperature. Males and females eggs respond differently, with females more sensitive to variation in incubation temperature. The different responses of both sexes have consequences on the sex ratio dynamic of hatchings with a weaker protandry after warm incubation. Temperatures experienced by eggs have more complex consequences on posthatching development. Later nymphal instars that hatched from eggs exposed to warm temperature were larger and bigger but developmental rate of the two populations was not affected. Our study demonstrates how incubation temperature could affect operational sex ratio and posthatching development in an insect and how this may be critical for population growth.

## Introduction

Fitness of ectothermic species is regulated through several life-history traits by temperatures at which juvenile or adult stages are exposed, either by direct or indirect effects, with consequences on their ecology (Huey and Berrigan 2001; Angilletta et al. 2009). Many studies show that temperature is the main factor that acts directly on insects (see a review in Bale et al. 2002). It could induce changes in development time, voltinism, population density, individual size, distribution, genetic composition, host plant exploitation, and insect/plant synchronization. All developmental stages, including eggs, are classically affected by their thermal environment (Howe 1967; Higaki and Ando 2002; Challet et al. 2005; Bonato et al. 2007), and eggs could respond early, as soon as the embryonic development starts. In species with winter diapausing eggs, the temperatures received during this stage are often crucial during their whole life cycle. Hence, numerous studies report effects of temperature exposure during the posthatching development of larvae (Nylin and Gotthard 1998), but prehatching temperatures affecting posthatching development is a novel idea for insects, documented so far in reptiles and birds (Shine 2004; Booth 2006; DuRant et al. 2010). Temperatures experienced during this egg stage may be crucial by directly affecting the mortality or the incubation time (Howe 1967), but could also have effects on posthatching life-history traits. Thus, egg diapause is regulated by environmental factors such as photoperiod and temperature (Tauber and Tauber 1976; Denlinger 2002) and the effect of chilling on the end of diapause and its consequences on insect emergence is well known in various orders (Collier and Finch 1983; Wipking 1995; Higaki and Ando 2002). Such effects of incubation temperature on others life-history traits, for example traits related to reproductive success as body size and weight, or population dynamic parameters, for example the time lag between male and female occurrence, are to our opinion poorly investigated and should receive more attention.

In this study, we examine how incubation temperature affects the hatching dynamics of males and females and posthatching development of nymphs in the leafhopper *Scaphoideus titanus* (Hemiptera: Cicadellidae). This insect is an important invasive species in Europe that transmits a phytoplasma disease, the Flavescence dorée from one grape to another (Schvester et al. 1969). *Scaphoideus titanus* is a native of the Great Lakes region in North America (Vidano 1966) and was reported for the first time in Europe in South Western France in 1958 (Bonfils and Schvester 1960), but we suspect an earlier invasion possibly some decades before (Chuche 2010). Now, *S. titanus* is spreading in Europe from ca. 35° to 50° N (Chuche and Thiéry 2009) and thermal conditions are supposed to explain its distribution. Considering its area of origin this insect is presumably well adapted to cold winter conditions. The southern limit of the leafhopper distribution is thus supposed to be due to the lack of cold temperatures that were initially supposed to be essential to break egg diapause (Caudwell and Larrue 1979; Steffek et al. 2007).

Here, we compare variation in *S. titanus* hatching dynamics, body size, weight, and population growth rate across a two incubation temperature. First, we submitted a field-collected population of eggs to artificial cold (5°C) or warm (20°C) three-month winter. Then, all eggs and hatching larvae were exposed to the same constant temperature in order to focus only on incubation. For both conditions, we examine the effects of temperature on several life-history traits to evaluate the relative fitness consequences of being exposed to cold versus warm temperature. We also hypothesize that the incubation temperatures induce variations of the operational sex ratio (OSR), which is the number of sexually active and ready males to available receptive females in the course of time of hatchings as a consequence of differential responses to temperature in each sex and affect fitness of nymphs.

## Materials and Methods

### Insects

The wild egg population was collected as previously described by Caudwell et al. (1970). Twenty-four kilograms of two-year-old grapevine woody canes (20–25 cm long) were collected in October 2008 from a vineyard without insecticide treatment in the southern Bordeaux area, where numerous *S. titanus* were observed during consecutive years as well as during the summer of 2008. A large number of canes was randomly collected by pruning the grapevine stocks at the end of October 2008, before the onset of winter and the exposure of the eggs to cold weather.

The presence of eggs was ascertained by examining a sample of 20 canes from which the bark had been carefully removed under a 45× microscope. Because no reliable method could be used to count the numbers of living eggs in the woody canes in order to ensure a similar egg number in each hatching cage, all the woody canes were randomized by grouping all the collected canes and separating them into 12 cages (50 × 38 × 36 cm) with ca. 2 kg in each. To avoid egg desiccation a 1 cm layer of vermiculite (Efisol, Nanterre, France) was placed on each cage floor, below the canes, and was moistened with distilled water spray every week.

### Thermal treatment

Hatching cages were placed in four different temperature-regulated chambers (PR-25T, ESPEC Corp., München, Germany). In two of them the eggs were submitted to a “warm winter” with a constant temperature of 20 ± 1°C; in the two others to a “cold winter” of 5 ± 1°C. For *S. titanus*, we considered temperatures equal to or lower than 5°C as cold. Because cold is a relative term and according to Salt (1961) which define cold as temperatures too low to support normal development of the insects concerned, the temperature 20°C was used to ensure eggs development. Moreover, this kind of temperature is common during winter in some South Spain vineyard region with no population of *S. titanus* yet observed, for example in Murcia (Brunet et al. 2008). The hatching cages were rotated within each climatic chamber once a week to minimize the effect of potential temperature gradients within the chamber.

After a three-month incubation, all the hatching cages were placed in one climatic chamber under a 16:8 (L:D) photoperiod, at 23 ± 1°C, and 65–70% relative humidity. In order to harvest neonate nymphs, six detached grapevine leaves (Cabernet-Sauvignon cultivar) kept in a glass tube with water were added to the cage ca. 20 days after the eggs were removed from the climatic chambers, and they were replaced when they began to wither (ca. every 15 days).

### Hatching dynamics and life-history traits

In each hatching cage, nymphs were gently removed each day from beneath the leaves using a pooter and the number of nymphs found was taken to be the number of hatching eggs. Observations ended when no more hatching occurred during seven consecutive days. The population hatching dynamics was determined using the daily counts of nymphs while the hatching dynamics for each gender was based on weekly data.

For life-history trait measurements, every week we isolated all the nymphs that emerged on Monday in a rearing cage (identical to the hatching cage) with two grapevine cuttings (Cabernet-Sauvignon cultivar) from the first cumulate number of hatching exceeding 100 and until this number dropped under this value. Depending on the duration of the hatching for the egg populations, we did this during nine weeks for eggs incubated at 20°C and during seven weeks for those incubated at 5°C. Thus, there were nine replicates of fitness measurements for 20°C and seven for the 5°C treatment. In each replicate, a sample of 40 insects was randomly selected and each insect was measured, until they become adults. Measurements were made to the nearest 0.01 mm using a micrometer under a stereomicroscope, from the head extremity without antennae to the telson ending; we also weighed fourth instar or older nymphs to the nearest 0.01 mg. We also checked the developmental instar and the gender from the fifth nymphal instar (Della Giustina et al. 1992). In order to compare the developmental rate of *S. titanus* egg populations incubated at 20 or 5°C, we treated all instars as equal development stages by averaging a population instar like the Developmental Index used by Bird and Hodkinson (2005) for Psyllidae:

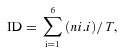

where *T*= total number of *S. titanus*, *i*= instar code (first nymphal instar = 1… fifth nymphal instar = 5, adult = 6), and *n_i_*= number of individuals in instar *i*.

### Statistical analysis

Hatching dynamics were compared with survival analysis because data are of the form “time until an egg hatch.” Survival analysis is usual in medical studies and many ecological studies produce such data that may be studied by these statistical tests (Muenchow 1986). Because there was no statistical difference between replicates for a same incubation temperature, replicates were pooled in a single hatching dynamic for each thermal treatment. Pair comparisons between hatching dynamics were performed with the log-rank and Gehan–Wilcoxon tests. The log-rank test accounts for all events throughout the period of observation, while the Gehan–Wilcoxon test puts more weight onto early events (Pyke and Thompson 1986). The former allows a general study of the dynamics, whereas the latter test is better to compare precocity of hatchings. A Cox proportional hazards model, a nonparametric multiple regression analog, was performed in order to determine the effect of egg's sex and temperature incubation on the hatching dynamics. Then, pairwise comparison was done with log-rank and Gehan–Wilcoxon tests.

Effects of instar, temperature, and replicate on size and weight were examined by using an analysis of variance (ANOVA) test after a logarithmic transformation in order to improve the normality of the data. Before performing the ANOVA tests, data were tested for normality using the Shapiro–Wilks test and homogeneity of variance using the Levene test. The best statistical model explaining the variation of the fitness parameters (size or weight) was determined with the Akaike information criterion (Akaike 1974). The homogeneity of variance between replicates allowed to make post hoc comparisons using a Tukey HSD test.

Developmental rate of leafhopper population issued from eggs incubated at 5 or 20°C were determined with linear regressions calculated with Index of development values. Regressions were then compared using Fisher's test (Snedecor and Cochran 1967).

All statistical analyses were performed using the software R 2.8.0 for Windows (R Development Core Team 2007).

## Results

### Hatching dynamics

**Figure 1:**
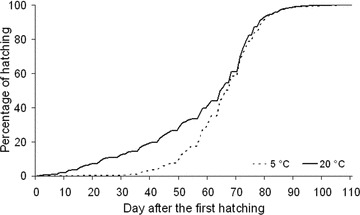
Cumulative percentage of hatchings after a three-month incubation at 5°C (broken line) and 20°C (solid line).

Cold temperature was not required to trigger hatching in our studied egg populations: eggs hatched both at 5 and 20°C (Fig. 1) in similar amounts (mean ± SD; 5°C = 1309.2 ± 160.5, 20°C = 1184 ± 166.4). However, temperatures clearly affected egg hatching dynamics: after incubation at 20°C it began four weeks earlier and lasted longer (107 days) than after incubation at 5°C (80 days) (Fig. 1; Log-rank: χ^2^= 99.4; Gehan–Wilcoxon: χ^2^= 255; both: *P* < 0.001).

**Figure 2:**
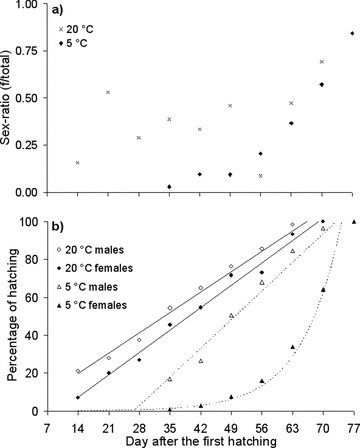
(A) Sex ratio (number of females/total) dynamics after incubation at 5°C and 20°C. (**B)** Cumulative percentage of males and females hatchings after a three-month incubation at 5°C and 20°C.

Incubation temperature did not affect the final sex ratio of hatchings of the overal egg population (0.35 at 5°C; 0.36 at 20°C), but interestingly it did change the evolution of sex ratio in the course of time (Fig. 2A). The sex ratio after incubation at 20°C, except for the last observation, was always close to 0.5 or male biased. Cold exposure of eggs led to an exponential increase of sex ratio of hatchings value that was male biased at the beginning and shifted to female biased at the end. Thus, the degree of protandry was weak after 20°C incubation and strong after incubation at 5°C.

Cox model showed that egg sex had no effect on hatching dynamic (χ^2^= 0.57; *P*= 0.45), contrary to incubation temperature dynamic (χ^2^= 59.84; *P* < 0.001). But, the sex:incubation temperature interaction had an effect (χ^2^= 59.97; *P* < 0.001). Indeed, the hatchings of males and females after warm or cold simulated winter were differentially affected. Males’ hatchings had a similar linear profile in both thermal conditions while female's profiles differed strongly (Fig. 2B). For a same gender, incubation temperature modified the hatching dynamics (Table 1). Males and females dynamics were different after a 5°C exposure, but were closest after warm incubation (Fig. 2B, Table 1). Actually, the global hatchings dynamics of both sex after 20°C incubation were similar and only the earliest hatching were different (Table 1). Thus, the different patterns of OSR after the two thermal treatments were mainly due to the different hatchings dynamics in each gender (Fig. 2B, Table 1). Hence, protandry varied with temperature incubation and the weaker degree of protandry observed after 20°C incubation was the consequence of similar hatching dynamics of males and females.

**Table 1:** Survival analysis (log-rank and Gehan–Wilcoxon tests) of males (m) and females (f) hatching dynamics from eggs exposed to cold (5°C) or warm (20°C) winter.

			20°C m	5°C f	20°C f
**5°C m**	Log-rank	χ^2^=	33.5	17.50	10.80
		*P*	<0.001	<0.001	<0.001
	Gehan–Wilecoxon	χ^2^=	56.10	18.00	26.00
		*P*	<0.001	<0.001	<0.001
**20°C m**	Log-rank	χ^2^=		35.20	3.17
		*P*		<0.001	0.053
	Gehan–Wilcoxon	χ^2^=		37.3	4.00
		*P*		<0.001	0.046
**5°C f**	Log-rank	χ^2^=			21.10
		*P*			<0.001
	Gehan–Wilcoxon	χ^2^=			27.00
		*P*			<0.001

### Life-history traits

**Figure 3:**
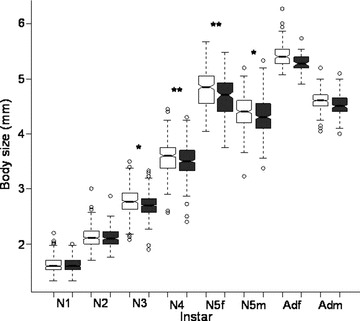
Body size of the different instars resulting from 5°C (solid boxplot) and 20°C (open boxplot) incubation. Nx, nymphal instar x; Ad, adult; f, female; m, male. *P < 0.05;**P < 0.01 (Tukey's HSD post hoc test).

**Figure 4:**
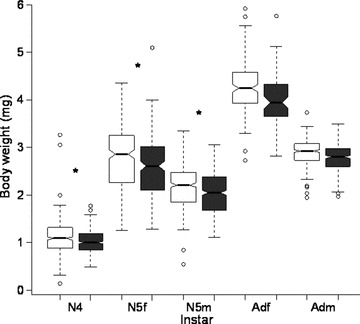
Body weight of the different instars resulting from 5°C (solid boxplot) and 20°C (open boxplot) incubation. Nx, nymphal instar x; Ad, adult; f, female; m, male. **P < 0.01 (Tukey's HSD post hoc test).

Posthatching development was also affected by incubation temperatures. Temperature acted on the body size of hatched insects (*F*= 57.18; *P* < 0.001) and there was an interaction with the instar (*F*= 3.39; *P* < 0.01). Third to five nymphal instars resulting from 20°C incubation were larger than from 5°C incubation (Fig. 3). As for size, temperature incubation had an effect on the weight (*F*= 87.37; *P* < 0.001) but there was no interaction between temperature incubation and developmental instar (*F*= 2.06; *P*= 0.08). Warm temperatures led to heavier nymphs than cold incubation (Fig. 4).

**Figure 5:**
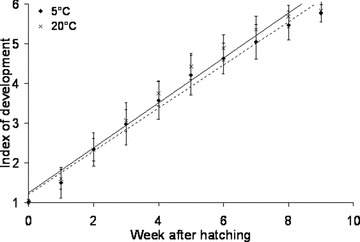
Evolution of the Index of development of *Scaphoideus titanus* from 5°C (broken line) and 20°C (solid line) incubation.

The developmental rate at the two populations could be presented by their linear regression (all *r*^2^ > 0.97, *P* < 0.001) (Fig. 5). There was no difference between the slopes for 5 and 20°C incubation temperatures (*F*= 0.34, *P* > 0.05). Thus, the incubation temperature did not affected the developmental rate.

## Discussion

Here, we examined if incubation temperature differently affects male and female hatching dynamics and posthatching development. We predicted that (1) OSR of hatching would be affected by the different responses to incubation temperatures of both sex and (2) fitness of individuals resulting from cold and warm winter would be different. Our results are in agreement with the first prediction but more mixed regarding the second. We found that OSR of hatching was seriously affected by incubation temperatures and resulted from a greater variation of female hatching dynamic rather than males. Concerning posthatching traits, development temperatures experienced by eggs had more complex consequences. Size and weight were affected but only later nymphal instars issued from eggs exposed to warm temperature were significantly larger and bigger. On the other hand, developmental rate of the two populations was not modified. We conclude that temperature at which insect eggs are exposed during incubation could have consequences on fitness through an action on posthatching development. By acting on critical parameters of population parameters, for example OSR, temperature incubation could have unsuspected impact on population growth and successful settlement of invasive species.

The main novelty of our study is the evidence that according to the incubation temperature of eggs, the degree of protandry was altered through the modification of female hatching dynamics. Our results demonstrate that male hatching patterns were constant whatever the incubation temperature, whereas variation was due to females. We show that an environmental factor to which diapausing eggs are exposed can affect protandry by acting only on one sex. To our knowledge, this effect of temperature had never been observed in insects before, but only in birds (Bogdanova and Nager 2008). Whether the difference in protandry is really due to plasticity and not to selection during overwintering remains an open-ended question. It should be mentioned that, because of similar observed levels of hatching, differential mortality rates could not explain the difference in protandry caused by the two incubation temperatures. We could logically suppose that, during the diapause maintenance phase (Kostal 2006), *S. titanus* eggs are not sensitive to temperatures that should then have no effect on development. However, during the postdiapause quiescence (Kostal 2006), development of embryos could resume with warm temperatures at a higher rate for females, while at cold incubation temperature female hatching are delayed longer than of males. Indeed, experiments on larvae show that external factors as temperature could affect differentially the growth rate of males and females and modulate protandry (Nylin and Gotthard 1998). The modification of the degree of protandry could have dramatic consequences on population dynamic. A bias in the occurrence of males and females over time affects the number of males and females that can breed at any time, and cost is often paid through reproductive failures (Grant et al. 1995; Clutton–Brock 2007) even if females could exhibit physiological and behavioral plasticity to reduce mating failures (Rhainds 2008). Indeed, mating success depend on the OSR (Grant et al. 1995; Clutton–Brock 2007). This ratio also predicts which sex will compete for mates and how intense this competition will be (Kvarnemo and Ahnesjo 1996). Moreover, protandry could avoid inbreeding by letting males disperse before the female emergence (Morbey and Ydenberg 2001). This is often observed when males are more mobile than females, which is the case in *S. titanus* (Clutton–Brock 2007). Thus, a poor degree of protandry between males and females could facilitate inbreeding and lead to decreased fitness. In our biological model, protandry does not completely disappear after incubation at 20°C. Assuming that males and females have similar growth rates, males that are smaller than females need less time to become adult (Nylin et al. 1993).

A question raised by our results concerning OSR is the relative importance of natural and sexual selection on protandry. Since Darwin (1871), protandry in insects is often explained as a sexually selected trait that favors mating of early emerging males. If protandry is due to sexual selection, selection on males would maximize mating and little sensitivity of protandry to environmental conditions. Indeed, if protandry is selected for per se through sexual selection in seasonal environments, environmental selection pressures do not act differentially on both sexes (Nylin et al. 1993). Protandry is not a static phenomenon and theoretically could evolve quickly (Bradshaw et al. 1997) and many studies assume that while the hatching pattern of females is genetically fixed the male hatching dynamics accept more plasticity, which allows variation in the degree of protandry. Our data demonstrate the contrary: male hatching patterns are similar whatever the incubation temperature, whereas those of females are radically different. We thus postulate that plasticity in the hatching dynamics regulation could concern not only the males, but more generally one gender or both according to species. In *S. titanus*, several traits required to produce protandry as a result of sexual selection are present: monovoltinism, monoandry, and polygyny (Lucchi et al. 2004; Chuche and Thiéry 2009). But, sexual selection cannot solely explain protandry in our case study, otherwise protandry should be maintained whatever the environmental conditions. We used a supposed homogenous eggs population for all the experiments that were subjected to two environmental conditions and this produced either two populations with very different degree of protandry. In our case study, even if protandry could maximize fitness via OSR this would be caused by a difference of sensibility of both genders to a natural factor (temperature). In this instance, natural selection did not select protandry as an adaptative advantage but could have maintained the difference of susceptibility to cold between males and females in order to optimize fitness.

Increased temperature during posthatching development generally results in higher growth rates, shorter developmental times, and smaller adult size in ectotherms, including insects (Sibly and Atkinson 1994). We found that increased incubation temperature also affects *S. titanus* fitness by inducing larger and bigger late nymphal instars. Such effect on posthatching development is well known in reptiles for ectotherms, in which body size, ratio of whole size to tails length, and hatchling locomotor performance increase with temperature incubation (Shine 2004; Booth 2006). Bird eggs can also respond to incubation temperature by providing bigger individuals after a warmer incubation (DuRant et al. 2010). Size and weight are usually positively correlated with reproductive success in insects (Nylin and Gotthard 1998), and weight is also positively correlated with flight performance and, thus, with dispersal for males (Marden 2000), with consequences on mobility and ability to find females (Tammaru et al. 1996). However, no significant differences were observed in *S. titanus* adults. Because this species is monovoltine with required diapause and that oviposition is almost impossible to observe in nature or on surrogates, we could not directly evaluate the reproductive success. The bigger body size and weight of late nymphal instars emerged from eggs incubated at 20°C did not correspond to longer developmental time, contrary to observed trade-off between juvenile development time and adult size in insects (Sibly and Calow 1986). Indeed, the developmental rate of the population at any time was the same for 20 and 5°C incubation. First larval instars and adults from the two incubation treatments have similar size and weight, so it is logical that developmental rates were similar even if intermediate instars show differences for these parameters.

In conclusion, winter temperatures to which eggs are exposed could deeply impact insect populations. We observed that high temperatures could enhance some life-history traits and decrease the degree of protandry, whereas colder temperatures have the opposite consequences. Egg incubation temperatures and their effect on protandry could explain the weak colonization of southern vineyards in Europe as well as supporting the hypothesis that winter temperatures affect the phenological synchrony between *S. titanus* and its host plants (Chuche and Thiéry 2009). By which mechanism temperature variation affects eggs of only one sex is still unresolved.
